# Severe Phenotype in Patients with Large Deletions of *NF1*

**DOI:** 10.3390/cancers13122963

**Published:** 2021-06-13

**Authors:** Laurence Pacot, Dominique Vidaud, Audrey Sabbagh, Ingrid Laurendeau, Audrey Briand-Suleau, Audrey Coustier, Théodora Maillard, Cécile Barbance, Fanny Morice-Picard, Sabine Sigaudy, Olga O. Glazunova, Lena Damaj, Valérie Layet, Chloé Quelin, Brigitte Gilbert-Dussardier, Frédérique Audic, Hélène Dollfus, Anne-Marie Guerrot, James Lespinasse, Sophie Julia, Marie-Christine Vantyghem, Magali Drouard, Marilyn Lackmy, Bruno Leheup, Yves Alembik, Alexia Lemaire, Patrick Nitschké, Florence Petit, Anne Dieux Coeslier, Eugénie Mutez, Alain Taieb, Mélanie Fradin, Yline Capri, Hala Nasser, Lyse Ruaud, Benjamin Dauriat, Sylvie Bourthoumieu, David Geneviève, Séverine Audebert-Bellanger, Mathilde Nizon, Radka Stoeva, Geoffroy Hickman, Gaël Nicolas, Juliette Mazereeuw-Hautier, Arnaud Jannic, Salah Ferkal, Béatrice Parfait, Michel Vidaud, members of the NF France Network, Pierre Wolkenstein, Eric Pasmant

**Affiliations:** 1 Service de Génétique et Biologie Moléculaires, Hôpital Cochin, DMU BioPhyGen, Assistance Publique-Hôpitaux de Paris, AP-HP, Centre-Université de Paris, F-75014 Paris, France; laurence.pacot@aphp.fr (L.P.); dominique.vidaud@aphp.fr (D.V.); audrey.briand@aphp.fr (A.B.-S.); audrey.coustier@aphp.fr (A.C.); theodora.maillard@aphp.fr (T.M.); cecile.barbance@aphp.fr (C.B.); beatrice.parfait@aphp.fr (B.P.); michel.vidaud@aphp.fr (M.V.); 2 Inserm U1016—CNRS UMR8104, Institut Cochin, Université de Paris, CARPEM, F-75014 Paris, France; ingrid.laurendeau@inserm.fr; 3 UMR 261, Laboratoire MERIT, IRD, Faculté de Pharmacie de Paris, Université de Paris, F-75006 Paris, France; audrey.sabbagh@parisdescartes.fr; 4 Inserm U1211, Service de Génétique Médicale, CHU de Bordeaux, F-33000 Bordeaux, France; fanny.morice-picard@chu-bordeaux.fr; 5 Department of Medical Genetics, Children’s Hospital La Timone, Assistance Publique des Hôpitaux de Marseille, F-13000 Marseille, France; sabine.sigaudy@ap-hm.fr; 6 Centre de Référence des Anomalies du Développement et Syndromes Malformatifs (UF 2970), CHU Timone, Assistance Publique des Hôpitaux de Marseille, F-13000 Marseille, France; olga.glazunova@ap-hm.fr; 7 Department of Pediatrics, Competence Center of Inherited Metabolic Disorders, Rennes Hospital, F-35000 Rennes, France; lena.damaj@chu-rennes.fr; 8 Consultations de Génétique, Groupe Hospitalier du Havre, F-76600 Le Havre, France; valerie.layet@ch-havre.fr; 9 Service de Génétique Clinique, CLAD Ouest, CHU Rennes, Hôpital Sud, F-35000 Rennes, France; chloe.quelin@chu-rennes.fr (C.Q.); melanie.fradin@chu-rennes.fr (M.F.); 10 Service de Génétique, CHU de Poitiers, F-86000 Poitiers, France; brigitte.gilbert-dussardier@chu-poitiers.fr; 11 Service de Neurologie Pédiatrique, CHU Timone Enfants, F-13000 Marseille, France; frederique.audic@ap-hm.fr; 12 Centre de Référence Pour les Affections Rares en Génétique Ophtalmologique, CARGO, Filière SENSGENE, Hôpitaux Universitaires de Strasbourg, F-67000 Strasbourg, France; helene.dollfus@chru-strasbourg.fr; 13 Medical Genetics Laboratory, INSERM U1112, Institute of Medical Genetics of Alsace, Strasbourg Medical School, University of Strasbourg, F-67000 Strasbourg, France; 14 Service de Génétique Clinique, CHU de Rouen, F-76000 Rouen, France; anne-marie.guerrot@chu-rouen.fr; 15 Service de Génétique Clinique, CH de Chambéry, F-73000 Chambéry, France; james.lespinasse@ch-metropole-savoie.fr; 16 Service de Génétique Médicale, CHU de Toulouse, Hôpital Purpan, F-31000 Toulouse, France; julia.s@chu-toulouse.fr; 17 Endocrinology, Diabetology, Metabolism and Nutrition Department, Inserm 1190, Lille University Hospital EGID, F-59000 Lille, France; Marie-Christine.VANTYGHEM@CHRU-LILLE.FR; 18 Dermatology Department, CHU Lille, University of Lille, F-59000 Lille, France; magali.drouard@chru-lille.fr; 19 Unité de Génétique Clinique, Centre de Compétences Maladies Rares Anomalies du Développement, CHRU de Pointe à Pitre, F-97110 Guadeloupe, France; marilyn.port-lis@chu-guadeloupe.fr; 20 Service de Génétique Médicale, Hôpitaux de Brabois, CHRU de Nancy, F-54500 Vandoeuvre-lès-Nancy, France; b.leheup@chru-nancy.fr; 21 Department of Medical Genetics, Strasbourg-Hautepierre Hospital, F-67000 Strasbourg, France; yves.alembik@chru-strasbourg.fr (Y.A.); alexia.lemaire@chru-strasbourg.fr (A.L.); 22 Bioinformatics Platform, Imagine Institute, INSERM UMR 1163, Université de Paris, F-75015 Paris, France; patrick.nitschke@parisdescartes.fr; 23 CHU Lille, Clinique de Génétique, Centre de Référence Anomalies du Développement, F-59000 Lille, France; florence.petit@chru-lille.fr (F.P.); anne.dieux@chru-lille.fr (A.D.C.); 24 Lille University, Inserm, CHU Lille, U1172 - LilNCog - Lille Neuroscience & Cognition, F-59000 Lille, France; eugenie.mutez@chru-lille.fr; 25 Department of Dermatology and Pediatric Dermatology, Bordeaux University Hospital, F-33000 Bordeaux, France; alain.taieb@u-bordeaux.fr; 26 Département de Génétique, APHP Nord, Hôpital Robert Debré, F-75019 Paris, France; yline.capri@aphp.fr (Y.C.); hala.nasser@aphp.fr (H.N.); lyse.ruaud@aphp.fr (L.R.); 27 UMR 1141, NEURODIDEROT, INSERM, Université de Paris, F-75019 Paris, France; 28 Department of Cytogenetics and Clinical Genetics, Limoges University Hospital, F-87000 Limoges, France; benjamin.dauriat@chu-limoges.fr; 29 Service de Cytogénétique et Génétique Médicale, CHU Limoges, F-87000 Limoges, France; sylvie.bourthoumieu@unilim.fr; 30 Department of Genetics, Arnaud de Villeneuve University Hospital, F-34000 Montpellier, France; d-genevieve@chu-montpellier.fr; 31 Département de Génétique Médicale et Biologie de la Reproduction, CHU Brest, Hôpital Morvan, F-29200 Brest, France; severine.audebert@chu-brest.fr; 32 Genetic Medical Department, CHU Nantes, F-44000 Nantes, France; mathilde.nizon@chu-nantes.fr; 33 Service de Cytogénétique, Centre Hospitalier Universitaire du Mans, F-72000 Le Mans, France; rstoeva@ch-lemans.fr; 34 Department of Dermatology, Reference Center for Rare Skin Diseases MAGEC, Saint Louis Hospital AP-HP, F-75010 Paris, France; geoffroy.hickman@aphp.fr; 35 Department of Genetics, FHU G4 Génomique, Normandie University, UNIROUEN, CHU Rouen, Inserm U1245, F-76000 Rouen, France; Gael.Nicolas@chu-rouen.fr; 36 Département de Dermatologie, Centre de Référence des Maladies Rares de la Peau, CHU de Toulouse, F-31000 Toulouse, France; mazereeuw-hautier.j@chu-toulouse.fr; 37 Département de Dermatologie, AP-HP and UPEC, Hôpital Henri-Mondor, F-94000 Créteil, France; arnaud.jannic@aphp.fr (A.J.); salah.ferkal@aphp.fr (S.F.); pierre.wolkenstein@aphp.fr (P.W.); 38 INSERM, Centre d’Investigation Clinique 1430, F-94000 Créteil, France

**Keywords:** neurofibromatosis type 1, NF1, *NF1* deletion, genotype–phenotype correlation, neurofibromas, NFs, malignant peripheral nerve sheath tumors, MPNSTs, tumor predisposition, learning disabilities, dysmorphism, skeletal abnormalities, cardiovascular abnormalities

## Abstract

**Simple Summary:**

Neurofibromatosis type 1 (NF1) is a genetic disorder caused by pathogenic variants in the *NF1* tumor suppressor gene. In 5–10% of NF1 patients, a large heterozygous deletion of the whole *NF1* gene is identified, leading to the commonly called “*NF1* microdeletion syndrome”. *NF1*-deleted patients were previously reported to develop a particularly severe form of the disease with frequent cognitive impairment and an increased risk of benign and malignant tumors. Here, we performed a comprehensive clinical assessment of the largest *NF1*-deleted cohort to date, including 126 NF1 patients with a deletion of the *NF1* gene. This work provides new insights into the specific phenotype associated with *NF1* deletions and may contribute to improve the follow-up care of NF1 patients.

**Abstract:**

Complete deletion of the *NF1* gene is identified in 5–10% of patients with neurofibromatosis type 1 (NF1). Several studies have previously described particularly severe forms of the disease in NF1 patients with deletion of the *NF1* locus, but comprehensive descriptions of large cohorts are still missing to fully characterize this contiguous gene syndrome. *NF1*-deleted patients were enrolled and phenotypically characterized with a standardized questionnaire between 2005 and 2020 from a large French NF1 cohort. Statistical analyses for main NF1-associated symptoms were performed *versus* an NF1 reference population. A deletion of the *NF1* gene was detected in 4% (139/3479) of molecularly confirmed NF1 index cases. The median age of the group at clinical investigations was 21 years old. A comprehensive clinical assessment showed that 93% (116/126) of *NF1*-deleted patients fulfilled the NIH criteria for NF1. More than half had café-au-lait spots, skinfold freckling, Lisch nodules, neurofibromas, neurological abnormalities, and cognitive impairment or learning disabilities. Comparison with previously described “classic” NF1 cohorts showed a significantly higher proportion of symptomatic spinal neurofibromas, dysmorphism, learning disabilities, malignancies, and skeletal and cardiovascular abnormalities in the *NF1*-deleted group. We described the largest *NF1*-deleted cohort to date and clarified the more severe phenotype observed in these patients.

## 1. Introduction

Neurofibromatosis type 1 (NF1; MIM#162200) is an autosomal dominant disease with a worldwide incidence estimated between 1 in 2500 and 1 in 3000 individuals [1]. In more than 95% of NF1 patients, a loss-of-function pathogenic variant is identified in the *NF1* gene (MIM*613113) [2,3]. *NF1* is a tumor suppressor located at 17q11.2; it contains 57 constitutive and three alternative exons and spans over ~350 kb. The 8454-nucleotide coding region (NM_00267.3) encodes a 2818-amino-acid protein, neurofibromin, which shows tumor suppressor functions [4] by negatively regulating the RAS-MAPK pathway [5]. About half of NF1 cases result from de novo pathogenic variants. 

NF1 diagnosis relies on the National Institutes of Health (NIH) criteria edited in 1988 [6]. These clinical criteria include multiple café-au-lait spots (CALS), axillary or inguinal freckling, Lisch nodules, optic pathway gliomas (OPGs), bone lesions, and neurofibromas (NFs). NFs are benign peripheral nervous tumors and are a pathognomonic manifestation of NF1. They may be cutaneous (cNFs), subcutaneous (scNFs), or plexiform (pNFs) neurofibromas. Plexiform neurofibromas are usually congenital or appear in early childhood in 30% to 50% of NF1 patients; pNFs present as a subtle enlargement of soft tissue with a “wrinkled” texture or a patch of hyperpigmentation with or without hypertrichosis. Although benign, pNFs are often life-threatening by their proximity to internal organs and can undermine quality of life. In ~10% cases, pNFs can transform into malignant peripheral nerve sheath tumors (MPNSTs) [7]. Other less specific clinical features can be observed in NF1 patients, such as musculoskeletal [8] or neurological [9] manifestations, cardiovascular malformations [10,11], endocrine alterations [12], cognitive behavioral disorders [13], Noonan-like features [14], or malignancies [15,16].

Despite a complete penetrance, NF1 displays a highly variable inter- and intrafamilial expressivity in its major features and in the occurrence of complications. Heritability studies have suggested the existence of a genetic component of the variable expressivity [17,18,19]. A large spectrum of pathogenic variations has been reported in the *NF1* gene [2,20,21], with 2783 unique variants reported in the Global Variome shared Leiden Open Variation Database (LOVD) on 19 February 2021. However, genetic studies have generally failed to establish clear-cut genotype–phenotype correlations according to the type of *NF1* pathogenic variant [2,22]. Recently, a study suggested an association between the presence of *NF1* truncating/splicing pathogenic variations or large deletions and the occurrence of “phenotypes requiring medical attention” [23]. International multicenter studies allowed the description of significant genotype-phenotype correlations for a few specific point pathogenic variants of the *NF1* gene. The first correlation was identified in 2007 and later confirmed in 2019 for the in-frame deletion of methionine 992 of neurofibromin [24,25]. The Met992 deletions resulted in a milder phenotype characterized by the quasi-absence of neurofibromas and OPGs but with an increased occurrence of Noonan-like features and pulmonic stenosis, when compared to the “classic” NF1 population. Similarly, missense variants affecting the arginine 1809 in neurofibromin were associated with significantly more frequent Noonan-like features, including pulmonic stenosis and short stature, and an increased tendency to develop cognitive impairment and/or learning disorders [26,27,28,29,30]. Arg1809 variants were also associated with an absence of NFs and OPGs. Absence of NFs was also observed in patients with missense variants affecting the methionine 1149, together with the presence of multiple CALS, skinfold freckling, and Noonan-like features [31]. More severe phenotypes were described for individuals with pathogenic missense variants of codons 844 to 848, located in the cysteine-serine rich domain (CSRD), or Arg1276 and Lys1423 in the GAP-related domain (GRD) of neurofibromin [31,32]. These patients suffered from more frequent pNFs (codons 844–848, Lys1423) or spinal NFs (codons 844–848, Arg1276), OPGs (codons 844–848), skeletal abnormalities (codons 844–848, Lys1423), and Noonan-like features including cardiovascular abnormalities (Arg1276, Lys1423).

Interestingly, a particularly severe presentation of NF1 was also evidenced in patients with large deletions encompassing *NF1* and several neighboring genes [33,34]. Patients suffered from a high burden of cutaneous NFs (cNFs) with an increased risk of developing MPNSTs. Clinical manifestations of *NF1*-deleted patients also typically included severe learning disabilities, and dysmorphic and Noonan-like features. *NF1*-deleted patients did not tend to have a short stature in adulthood, unlike *NF1*-mutated patients [35].

The emergence of genomic medicine, linked to the development of next generation sequencing (NGS) technologies 10 years ago, has enabled a more precise and rapid molecular characterization of many NF1 patients. These advances have led to the description of a growing number of *NF1* pathogenic variants. Cohort analysis has provided the description of a “classical” NF1 phenotype, a necessary basis for the identification of genotype–phenotype correlations. In the present study, we aimed to confirm the distinctive phenotype associated with large deletions of the *NF1* locus.

## 2. Materials and Methods

### 2.1. Study Cohort

Databases from routine molecular diagnosis at the Cochin Hospital, Paris, France, between 2013 and 2020 were retrospectively analyzed. Between 2013 and 2020, 4091 index cases (IC) with a clinical presentation suggestive of NF1 were molecularly screened for NF1 in the Cochin Hospital (APHP, Paris, France): 2875 index cases (IC) were confirmed with an *NF1* pathogenic variant. In addition, 648 NF1 patients were enrolled between 2005 and 2013 in two French clinical research programs (*Programme Hospitalier de Recherche Clinique*, PHRC) entitled ‘Patients at risk of scalability in neurofibromatosis type 1: phenotypic, genotypic and proteomic comparative study in a cohort’ (PHRC ‘*EvoNF*’; 2005–2008; inclusion of 219 unrelated adult NF1 patients) and ‘Study of the expressivity of neurofibromatosis 1: identification of modifier genes’ (PHRC *GenModif* 2010–2013; inclusion of 429 unrelated adult NF1 patients) as part of the ‘NF-France database’ [19]. In these two programs, a pathogenic variant of the *NF1* gene was identified in 604 index cases. All patients gave their written informed consent.

### 2.2. Phenotypes

Phenotypical data were recorded with a standardized questionnaire including biometrics and the main clinical features observed in NF1. When clinical assessment was missing or incomplete in the laboratory databases, referent clinicians were contacted to obtain additional clinical data recorded during the follow-up of their patients. Patients with missing data were considered as ‘not specified’ (NS) for the trait and were not included in the statistical analysis for that trait. CALS were counted regardless of their size. Most features were identified by physical examination, with Lisch nodules being diagnosed, or excluded, by slit-lamp examination; individuals not given a slit lamp examination were coded as ‘not documented’ (ND) and excluded from further analysis of the trait. The presence or absence of OPGs was determined by cranial MRI or CT examination, with individuals not given cranial imaging being encoded as ‘ND’. Facial dysmorphism was evaluated on the following aspects: coarse face, flat occiput/brachycephaly, facial asymmetry, prominent forehead, frontal bossing, ptosis, hypertelorism, midface hypoplasia, triangular face, small and down-slanting palpebral fissures, eversion of the lateral eyelid, epicanthic folds, large and low set ears, high and broad nasal bridge, bulbous nasal tip, hooked nose, wide and prominent philtrum, micrognathia, small pointed chin, low posterior hairline, webbed neck. Stature and weight were evaluated according to the 2018 AFPA (*Association Française de Pédiatrie Ambulatoire*) reference curves. Head circumference was evaluated according to the 2018 AFPA reference curves for children under 5 years old and according to the Nellhaus charts when older. Short and tall stature were defined as a height that was more than 2 standard deviations (sd) respectively below or above the age- and sex-matched population mean. Macrocephaly was defined as a head circumference that was more than 2 sd above the age- and sex-matched population mean. The diagnosis of learning disabilities was performed on specific testing of cognitive abilities and/or history of scholar difficulties leading to repeating at least one level. As the small number of observations of each individual malignancies would have resulted in a lack of power in statistical analyses, we pooled all types of malignant tumors into a single item (namely “malignancies”) and performed analyses on this composed trait.

### 2.3. NF1 Molecular Analysis

The molecular analysis of the *NF1* gene was performed using a variety of screening methodologies including DNA and RNA sequencing, polymorphic microsatellite (MS) marker analysis, and multiplex ligation-dependent probe amplification (MLPA), comparative genomic hybridization array (CGHa), or real-time PCR-based gene-dosage analysis to permit deletion assessment, as previously described [36,37,38]. NGS experiments were performed at the NGS facility of Cochin Hospital, Paris (Assistance Publique-Hôpitaux de Paris AP-HP, France). A custom-made amplification panel (Thermo Fisher Scientific, Courtaboeuf, France) and an Ion S5 XL system (Thermo Fisher Scientific) were used to sequence the coding and IVS flanking (25bp) regions of the *NF1* and *SPRED1* genes. The sequence analysis was performed according to the Genome Analysis Tool Kit (GATK) guidelines using Polyquery (Paris Descartes University, Paris, France). Assessment of variants implication was performed based on population databases (dbSNP and gnomAD), variant databases (HGMD, LOVD, and COSMIC), and predictions softwares (Alamut and mutation taster). An assessment of variants’ pathogenicity was performed according to the American College of Medical Genetics and Genomics and the Association for Molecular Pathology (ACMG-AMP) guidelines [39]. Copy number variation analysis (CNA) was performed using the quantitative values obtained from the number of reads for each amplicon of each sample. For *NF1* CNA analysis, *SPRED1* was considered as the control gene, and reciprocally for the *SPRED1* CNAs. Read number for each *NF1* and *SPRED1* amplicon was normalized by dividing each amplicon read number by the total of amplicon read numbers of a control gene from the same sample. Copy number ratios of <0.7 and >1.3 were considered deleted and duplicated, respectively.

### 2.4. Statistical Analyses

Univariate analysis was performed using a two-tailed Fisher’s exact test to compare categorical variables. Resulting *p* values were adjusted using the Benjamini-Hochberg (B-H) correction for multiple comparisons [40]. Reference cohort for comparison (“classic NF1”) was obtained from Koczkowska et al. [31]. Statistical analyses were performed with the R stats package in RStudio v4.0.3. Radar charts were obtained with the fmsb package in RStudio v4.0.3.

### 2.5. Characterization of the Deletions

The SALSA MLPA P122 NF1-area (MRC Holland, Amsterdam, Netherlands) was used to determine the *NF1* deletion type in patients: type 1, type 2, type 3, or atypical deletions, as previously described [41]. Briefly, 100 ng of patients’ DNA were denatured at 98 °C and hybridized to the kit probes at 60 °C. Ligation was performed at 54 °C, followed by an amplification step with 35 PCR cycles. Fragment separation and detection using GS-500LIZ marker were performed on ABI Prism 3130 automatic DNA sequencer (Thermo Fisher Scientific). The results were analyzed with the GeneMapper^®^ v.4.0 software package (Thermo Fisher Scientific). The kit contains ten control probes on autosomes, one on X, and one on Y chromosomes. Twenty-three probes are distributed along the 17q11.2 region (5 *NF1*-intragenic, 11 centromeric, and 7 telomeric) and two are located in *ASPA* (17p13.2) and *PMP22* (17p12) genes, respectively. Deletions encompassing *SUZ12P1* to *LRRC37B* probes were classified as type 1, and those including probes in exon 1 of *NF1* to *LRRC37B* were classified as type 3. Type 2 deletions included at least probes from *CRLF3* to *UTP6*, and probes in *SUZ12* and/or *SUZ12P1* to different degrees. Other cases were classified as atypical deletions.

## 3. Results

### 3.1. Description of the Cohort

Between 2013 and 2020, 4091 index cases (IC) with a clinical presentation suggestive of NF1 were molecularly screened for *NF1* in the Cochin Hospital. Pathogenic variants for the gene were detected in 2875, among which 114 were deleted. In addition, between 2007 and 2013 in the two French clinical research programs *EvoNF* and *GeneModif*, 648 IC were tested for *NF1* variant. In 604, a *NF1* pathogenic variant was identified, including 25 patients with a whole *NF1* gene heterozygous deletion. Altogether, deletions of the *NF1* locus represented 4% (139/3479) of positive IC of the cohorts. Patients who refused to be included in research programs or with insufficient clinical data were excluded from the present study. A total of 126 patients were enrolled in this study, with 122 index cases.

Patients ranged between 4 months and 69 years old, with a median at 21 years old (Table 1, Figure S1) and a mean at 22. Forty-five percent (57/126) of the individuals were less than 18 years old. Forty percent (51/126) were males. About 92% (116/126) of all patients fulfilled the clinical NIH criteria, among which the most frequent were: the presence of (i) 6 or more CALS, (ii) axillary or inguinal freckling, and (iii) 2 or more NFs or one pNF (Figure S2). Ninety-one percent (84/92) of patients for whom quantitative data was available had 6 or more CALS. Although about 50% (52/107) of patients had musculoskeletal abnormalities, only 7% (8/107) showed bone lesions such as sphenoid dysplasia, tibial pseudarthrosis or dystrophic scoliosis. Among the 9 patients not fulfilling the NIH criteria for NF1, two were more than 18 years old: a 62-year-old female had a late-onset NF1 and started to notice the presence of neurofibromas around 35 years old; the other adult patient was a 46-year-old woman without any familial history of NF1. She presented with several cutaneous neurofibromas and learning disabilities.

#### 3.1.1. Pigmentary Manifestations

Almost all patients with deletion of the *NF1* locus presented with CALS (99%; 122/123). The only patient with no detectable CALS had more than 100 cNFs that may have rendered the research for other cutaneous manifestations complex. She also had Lisch nodules evidenced by slit lamp examination. Lisch nodules were predominantly observed in adults, with an increased prevalence with age as previously described (17%, 3/18, before 9 years old; 40%, 6/15, between 9 and 18; 70%, 31/44, in adults) [42]. Other cutaneous findings included xanthogranuloma (*n* = 1), birthmark (*n* = 1), verrucous nevus (*n* = 1), and anemic nevus (*n* = 1). One 3-year-old patient had hypertrichosis. Blue–red macules were not systematically searched for during the follow-up of the patients, although it constitutes a precocious sign of the disease [43]. They were evidenced in 20% (15/74) of patients.

#### 3.1.2. Neurofibromas

In the present cohort, 69% (84/121) of the patients had at least two cNFs or scNFs or one pNF. Among the remaining 31% (37/121), 5/37 patients were more than 18 years old. Most underage individuals did not receive an MRI examination; thus, the existence of asymptomatic pNFs could not be excluded. Fifteen patients were massively affected and had more than 100 cNFs all over the body, in association with scNFs in 10 cases and with pNFs in 5 cases. pNFs were identified in 23% of patients for whom data was available (17/75) (Table 1). They were never isolated and were most often associated with more than 10 cNFs and/or scNFs. One teenage patient received a treatment with trametinib against multiple deep and spinal NFs resulting in spinal cord compression, walking difficulties and neuropathic pain. Another adult patient underwent multiple excisions of bulky cNFs and of two spinal NFs. Symptomatic spinal NFs were generally associated with back pain or motor deficits.

#### 3.1.3. OPGs and Neurological Findings

OPGs were identified in 11% (9/80) of patients and were mainly diagnosed in underage patients. They resulted in loss of visual acuity in four patients, with one presenting bilateral optic atrophy.

The other main neurological finding in the population of individuals with a deletion of the *NF1* gene was the presence of unidentified bright objects (UBOs) on cranial MRI (56%, 35/62). These UBOs were virtually detected in every region of the brain, with a predominance in cerebellum and cerebellar peduncles (*n* = 14), basal ganglia (*n* = 7), and thalamus (*n* = 6).

Two individuals suffered from left hearing loss. One was congenital and due to ear canal stenosis with a deformation of the squama.

Learning disabilities or cognitive impairments were present in 73% (74/102) of patients and were found in association with language delay (*n* = 12), global developmental delay (*n* = 9), attention deficit disorders (*n* = 7), psychomotor delay (*n* = 5), hyperactivity (*n* = 4), behavior disorders and self-aggressiveness (*n* = 3), and dysgraphia (*n* = 2).

#### 3.1.4. Malignancies

In the present cohort, 12% (10/84) of patients developed a cancer, including a 12-year-old girl and three adults who developed MPNSTs. A 35-year-old woman without malignancy had a family history of a sister who died from an MPNST and could not be included in the present study. Three patients developed a breast cancer, of which one was less than 50 years old.

#### 3.1.5. Cardiovascular Abnormalities

Cardiovascular abnormalities concerned 23% (15/64) of *NF1*-deleted patients and included hypertension (*n* = 7), pulmonic stenosis (*n* = 2), aortic stenosis (*n* = 1), extra-dural hematoma (*n* = 1), patent ductus arteriosus (*n* = 1), septal hypertrophy (*n* = 1), aberrant vessel in ascending aorta (*n* = 1), and occlusion of the left internal carotid artery (*n* = 1). The presence of pulmonic stenosis was not ascertained in the 25 patients from the two PHRC.

#### 3.1.6. Other Findings

As short stature is frequently reported in NF1 patients, we investigated that in our cohort. We observed that 1% (1/77) of patients was more than 2sd taller than the mean age- and sex-matched general population, 19% (15/77) at +1sd, 65% (50/77) at mean, 11% (8/77) at −1sd, 3% (2/77) at −2sd, and 1% (1/77) at −3sd. Co-occurrence of tall stature and OPGs was not observed in our population [44]. Deep palmar and plantar creases were identified in two patients, with excess of skin in one of them. Ten patients showed hyperflexibility of joints.

Two patients were diagnosed with hypothyroidism. Additionally, one had a goiter, and one had a surgical removal of a neurofibroma of the thyroid gland. One patient had a keratoconus.

Co-occurrence of NF1 and other genetic diseases within the same family was noticed in three cases: one 15-year-old patient had concomitant familial polycystic kidney (this condition was already described and genetically confirmed in [45]); another patient inherited Charcot-Marie-Tooth disease type 1A from her mother, a condition that was already suggested or observed in the past [46,47,48,49]; also, one patient had a daughter diagnosed with Costello syndrome but no NF1.

### 3.2. Comparison of the Clinical Findings in the French NF1-Deleted Cohort with “Classic NF1” Phenotype

**Figure 1:**
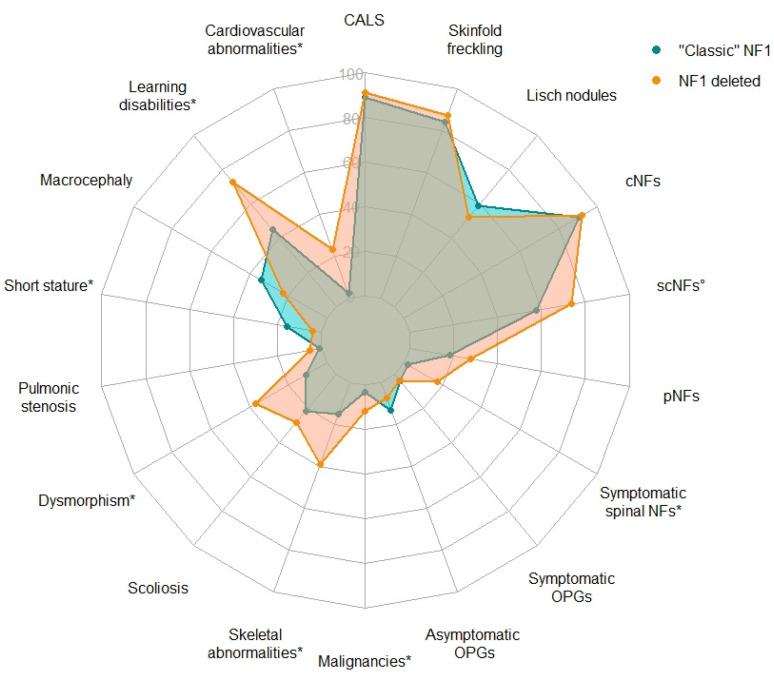
Radar chart of the frequency of the main clinical findings in the French *NF1*-deleted cohort and in “classic” NF1. Frequency of features with a white circle (°) or an asterisk (*) significantly differ between the two cohorts, respectively only before or after correction for multiple testing with the Benjamini-Hochberg (B-H) procedure. CALS: café-au-lait spots; cNFs: cutaneous neurofibromas; OPGs: optic pathway gliomas; pNFs: plexiform neurofibromas; scNFs: subcutaneous neurofibromas.

Statistical comparisons of the main clinical findings in NF1 versus the reference “classic” NF1 population are summarized in Table 2 and Figure 1; *p* values were adjusted using the Benjamini-Hochberg (B-H) correction for multiple comparisons.

For clinical features that usually develop during adolescence or adulthood (cNFs, scNFs, scoliosis), only patients >18 years old were considered for statistical analyses. Benign pigmentary lesions were not significantly more frequent in the *NF1*-deleted group than in the “classic” NF1 population. We observed a significantly higher proportion of individuals with scNFs and spinal NFs in the *NF1*-deleted patients. The presence of scNFs, particularly when they are in great number, have previously been associated with the development of internal neurofibromas at risk for malignant transformation in NF1 patients [50]. Related to this observation, malignant tumors were significantly over-represented in the *NF1*-deleted group (*p* value = 0.0072 after correction for multiple testing). As pNFs mostly grow in young children [51], we focused on individuals >8 years-old and considered all types of pNFs. We observed a tendency towards a higher number of pNFs in the *NF1*-deleted patients, though not significant (*p* value = 0.089 and 0.13 respectively before and after correction).

Skeletal abnormalities were twice as frequent in *NF1*-deleted patients as in the general NF1 population (42/107 *vs.* 144/948). However, it must be taken into consideration that we included more clinical features in that category than previous studies that mainly focused on scoliosis, pseudarthrosis, or long bone dysplasia. As previously described in [34], we confirmed the higher prevalence of dysmorphism and learning disabilities in *NF1*-deleted patients (adjusted *p* values 6.3 × 10^−7^ and 2.2 × 10^−6^, respectively).

An important difference was observed for the cardiovascular abnormalities which were identified in >23% of *NF1*-deleted patients versus less than 3% for the general NF1 group (15/64 *vs.* 54/2322, adjusted *p* value = 1.7 × 10^−9^). Pulmonic stenosis was found in 5% (2/39) of *NF1*-deleted patients, but the group for which cardiovascular issues were assessed was too small to achieve significance against the reference cohort (adjusted *p* value = 0.13).

### 3.3. Characterization of the Deletions of the NF1 Locus

MLPA analysis of the *NF1* locus was performed in the DNA samples from 108 *NF1*-deleted patients. Among them, 66% (71/108) carried type-1, 19% (21/108) carried type-2, and 5% (5/108) carried type-3 deletions (Table S1). Atypical profile was observed in 11 samples (10%). A significantly higher proportion of scNFs, pNFs, skeletal abnormalities, dysmorphism, cognitive impairment, learning disabilities, and cardiovascular abnormalities were observed in type-1 deleted patients when compared to the “classic” NF1 group. The occurrence of malignancies did not achieve significance anymore in any category, as DNA samples from patients with MPNST (*n* = 2), breast cancer (*n* = 2) and intestinal tumor (*n* = 1) were either insufficient or of low quality and could not allow the classification of these patients. Non-Hodgkin lymphoma was observed in a type-2 deleted patient, but no causality of the *NF1* deletion was established.

## 4. Discussion

Phenotypical variability in NF1 is a challenge for health professionals and may complicate genetic counselling. Recent advances in the NF1 field and large international multicenter studies have broadened our knowledge on the NF1 clinical expressivity. Publications in the last ten years have led to the identification of rare but specific genotype–phenotype correlations: variations of three codons of neurofibromin (Met992, Met1149, Arg1809) were shown to be associated with milder forms of NF1, while pathogenic variants in seven other codons (codons 844–848, Arg1276, Lys1423) located in functional domains of the protein were associated with more severe symptoms (Table S2) [24,25,26,27,28,29,30,31,32]. A more severe presentation of patients with complete deletions of the *NF1* gene was also suggested in past studies [33], but only two investigations in large well characterized NF1 cohorts were previously conducted [34,52].

In the present study, we clinically described the largest *NF1*-deleted cohort including 126 patients with molecularly confirmed heterozygous deletion of the *NF1* gene. Pigmentary manifestations of NF1 mainly include CALS, skinfold freckling and Lisch nodules. All three are benign and do not cause any functional disturbance. They originate from the deregulation of melanogenesis in melanocytes [53] as a result of activation of the cAMP-mediated PKA and ERK1/2 signaling pathways [54]. We observed that more than half of the *NF1*-deleted patients had café-au-lait spots, skinfold freckling, Lisch nodules, neurofibromas, neurological abnormalities, and cognitive impairment or learning disabilities. Musculoskeletal abnormalities were diagnosed in 49% (52/107) of patients, mainly implying scoliosis (25/107), hyperflexibility of joints (10/82), and pectus abnormalities (9/82). Though the two latest seem to be more specific to deleted patients [52], scoliosis is a frequent outcome in the “classic” NF1 population [55]. However, scoliosis seems to be more frequent in underage *NF1*-deleted patients, though not significantly (8/48 *vs.* 2/39 [56], *p* value = 0.183). UBOs were frequent in our study cohort but they were also reported in up to 94% of a “classic” NF1 population [57]. UBOs appear as hyperintense lesions on T2-weighted images, but their clinical significance is uncertain and their association with neurocognitive functions remained elusive [58]. Their number and size tend to increase until the age of 10 years old and then to spontaneously regress over time [59]. UBOs are a non-specific but early sign of the disease, like CALS and skinfold freckling which can be found in Legius syndrome or other syndromes [60] but also in the general population [61]. UBOs could be considered as a good candidate criterion for the diagnosis of NF1 in children.

Neurofibromas constitute the pathognomonic symptom of NF1. However, as for the case of Lisch nodules, cNFs and scNFs mainly appear after the first decade of life [62]; pNFs can be earlier and even appear during the neonatal period. In our study, cNFs and scNFs were more frequent in adults (59/64 and 39/53, respectively) than in children under 8 years old (2/31 and 3/27) or in patients between 9 and 18 years old (10/19 and 11/14). Although most studies did not provide detailed information about underage NF1 patients, two of them were of interest to explore the prevalence of cNFs and scNFs [56,63]. In Huson et al., the proportion of cNFs was estimated at 10% before 11 years old, and at 57% between 11 and 20 years old. In McGaughran et al., the estimation was at 23% before 10, and at 47% between 10 and 19 years old for cNFs, and at 13% before 10 and 40% between 10 and 19 years old for scNFs. Although the age ranges were slightly different, these data are comparable to what we observed in our cohort for cNFs: 2/31 (6%) before 9, and 10/19 (53%) between 9 and 18 years old. For scNFs, they were also in line with what we observed in patients before 9 (3/27: 11%) and confirm the higher prevalence of scNFs in our *NF1*-deleted cohort between 9 and 18 years old (11/14: 79%). We observed that 28% (17/61) of patients over 8 years old for which the trait was evaluated presented with pNFs, either externally visible or assessed by MRI. Previously described cohorts used as a reference for pNFs (“classic” NF1) only focused on externally visible pNFs (18%, 120/648), which could argue for the discrepancies observed in the two groups. However, it might be of interest to explore further for a possible higher risk or an earlier appearance of pNFs in *NF1*-deleted patients. In our cohort, symptomatic spinal neurofibromas were significantly more frequent in the *NF1*-deleted patients (6/35 *vs.* 36/2,058; *p* value = 1.7 × 10^−4^ after correction for multiple testing). Altogether, *NF1*-deleted patients had a higher tumor burden than non-deleted NF1 patients [64].

The most frequent central nervous system (CNS) tumors observed in NF1 are gliomas which concern about 20% of patients [65]. They are mainly low-grade gliomas of the optic pathways and are most often classified as pilocytic astrocytoma [66,67,68]. In our cohort, OPGs were present in 11% (9/80) of patients and were responsible for loss of visual acuity in three patients, with one presenting bilateral optic atrophy. NF1 also predisposes to a wide spectrum of malignancies, of which MPNSTs and brain tumors are the most represented [55]. *NF1*-deleted patients would be particularly at risk of developing malignancies, especially MPNSTs [33] which occurred in 5% (4/84) of patients in the present study. This prevalence may be underestimated in our cohort showing a median age of 21, with 45% (57/126) of patients being minor at the time of clinical investigation. Only long-term follow-up in prospective studies could give a definitive confirmation of an increased MPNST risk in *NF1*-deleted patients. In our cohort, breast cancer was diagnosed in three patients, of whom one was less than fifty years old. A previous work suggested that *NF1* deletions would not be responsible for an increased risk for breast cancer as no cases were observed in patients with *NF1* deletions in this report [69]. These results are not corroborated by our study. Reported malignancies also included a non-Hodgkin lymphoma (*n* = 1), an intestinal tumor (*n* = 1), and a thoracic synovial sarcoma (*n* = 1). Reported malignancies in the “classic” NF1 group included MPNSTs (*n* = 15), chronic myeloid leukemia (*n* = 1), rhabdomyosarcoma (*n* = 1), and breast cancers (*n* = 2), with some patients developing several malignant or non-malignant tumors.

Cardiovascular malformations have been reported in many patients with NF1 [10,11], but overall frequency of these findings in *NF1*-deleted patients had never been evaluated before. Pulmonic stenosis is the most frequently reported congenital heart disease in NF1 and is almost always found in association with Noonan-like features [20], as it is the case in our *NF1*-deleted group (*n* = 2/2 patients with pulmonic stenosis and dysmorphic features). Altogether, cardiovascular abnormalities were frequent in our group (23%, 15/64) and included hypertension (*n* = 7), pulmonic stenosis (*n* = 2), aortic stenosis (*n* = 1), extra-dural hematoma (*n* = 1), patent ductus arteriosus (*n* = 1), septal hypertrophy (*n* = 1), aberrant vessel in ascending aorta (*n* = 1), and occlusion of the left internal carotid artery (*n* = 1). Previous studies also identified ventricular septal defect, atrial septal defect, aortic stenosis, mitral valve prolapse or insufficiency, and hypertrophic cardiomyopathy [33].

While NF1 patients are usually described with a short stature [70,71], *NF1*-deleted populations are depicted to be taller [35], with large hands and feet. The proportion of short stature patients was significantly lower in our deleted cohort when compared to the “classic” NF1 population (3/77 *vs.* 109/684, *p* value = 0.0086 after correction for multiple testing). In comparison to previously reported NF1 populations, we observed a significantly higher proportion of symptomatic spinal neurofibromas, dysmorphism, learning disabilities, malignancies, and skeletal and cardiovascular abnormalities in the present *NF1*-deleted cohort. These results are in line with previous observations [33,34] and confirm the overall more severe presentation of the disease in *NF1*-deleted patients. 

The majority of NF1-associated symptoms (including pigmentary lesions, pseudarthrosis, neurofibromas, and other tumors) are due to the complete loss-of-function of the *NF1* gene (resulting from the somatic inactivation of the *NF1* wild-type allele) [54,72,73,74]. Thus, constitutional loss of the *NF1* gene alone may not explain the vast spectrum of variable expressivity observed in *NF1*-deleted patients. In *NF1*-deleted patients, *NF1* locus deletion generally encompasses not only the gene itself, but also its flanking genes [75]. Three different types of recurrent deletions of the *NF1* locus were previously described; they implied low-copy repeats (LCRs) located at 17q11.2 region: *NF1-REP-c* and *NF1-REP-a* or *NF1-REP-b* (for type 1 and type 3 deletions, respectively) or *SUZ12* and its pseudogene *SUZ12P* for type 2 deletions [33,34,76]. These deletions vary in size (approximately 1.4 Mb for type 1, 1.2 Mb for type 2, and 1 Mb for type 3 deletions) and in the number of deleted genes. These recurrent deletions of the *NF1* locus were suggested to be responsible for a contiguous gene syndrome. Loss of *SUZ12* in type 1 and type 2 deletions may promote MPNST development in *NF1*-deleted patients through its role in the polycomb repressive complex 2 (PRC2): recent advances in genomic studies of MPNSTs identified critical involvement of PRC2 core components SUZ12 and EED in transition to malignancy [77,78,79]. Further functional studies are needed to provide confirmation of the other phenotypes suggested to be more often associated with large deletions of the *NF1* locus. Interestingly, haploinsufficiency of the *ADAP2* gene has been suggested as causal for the increased cardiovascular abnormalities found in *NF1*-deleted patients. Morpholino-mediated knockdown of *adap2* in zebrafish embryos resulted in variable circulatory defects at two days postfertilization, caused by abnormal heart development and function [80]. Even less frequent, atypical *NF1* microdeletions with non-recurring breakpoints have also been reported [33]. Molecular characterization of large prospective cohorts would be needed to delineate the specific implications of each subtype of *NF1* locus deletions and to specify the role of the different deleted genes on the clinical outcomes and phenotypes of deleted patients.

We noticed a high prevalence of sporadic cases in our cohort (103/119, 87%), consistent with our previous descriptions [34]. In the cohort, three patients showed *NF1* mosaic deletions with 30–40% allelic frequencies (variant allele frequency, VAF) estimated by NGS and confirmed by MLPA. The first patient was a 46-year-old woman. MLPA on extracted blood DNA gave an estimate of 30% VAF type-1 deletion of the *NF1* locus. Clinically, she had few CALS, unilateral Lisch nodules, multiple cNFs limited to the arm and the head, two deep neurofibromas, cervical scoliosis, large hands and feet, and had personal history of psychotic episodes and ductal carcinoma *in situ* of the breast. The second patient with mosaic NF1 was a 23-year-old woman with type-2 deletion. She presented with a few CALS and freckling, bilateral Lisch nodules, symptomatic OPG, UBOs on MRI, headaches, learning disabilities, and a precocious puberty. She also had one scNF with no histological confirmation. The third mosaic patient was a type-2 *NF1*-deleted 2-year-old girl with only pigmentary manifestations (CALS, freckling, and Lisch nodules). MLPA analysis of the *NF1* locus suggested a fourth case of mosaicism in a 24-year-old man. Allelic frequency of the type-2 deletion was estimated around 40% (ratios 0.58 to 0.63 between the *CRLF3* and *UTP6* deleted probes). The patient presented CALS, freckling and Lisch nodules. Altogether, at least 3 of the 21 type-2 *NF1* deletions (14%) were post-zygotic alterations. The frequent occurrence of de novo *NF1* deletions recommends that potential somatic mosaicism should be considered when performing phenotypical assessment.

## 5. Conclusions

In conclusion, the retrospective study of a large cohort of patients with complete deletion of the *NF1* locus (the most frequent genetic alteration found in NF1 patients) allowed us to confirm the association between *NF1* deletion and a particularly severe form of the disease. These results may help in adapting medical care and genetic counselling for these patients. Resorting to imaging techniques should be encouraged in this at-risk population when confronted with an unusual symptomatology. Our study also suggests the need to implement molecular techniques allowing the detection and sizing of the *NF1* locus deletions, which will make it possible to refine the genotype–phenotype correlations related to the large deletions in NF1 patients.

## Figures and Tables

**Table 1:** Clinical findings in the *NF1*-deleted cohort.

Clinical Features	0–8 Years Old	9–18 Years Old	>18 Years Old	All Patients n/N *	All Patients %
Age range	4 months–8 yo	9–18 yo	18–69 yo	4 months–69 yo	
Median age (years)	5	13	32	21	
Number of individuals (proband:relative)	31:3	22:1	69:0	122:4	
Male:Female	16:18	12:11	23:46	51:75	
Fulfilling the NIH criteria if the family history is taken into account	28/34	21/23	67/69	116/126	92%
Fulfilling the NIH criteria if solely taking the physical signs into account	27/34	21/23	67/69	115/126	91%
CALS ^a^	34/34	22/22	66/67	122/123	99%
1–5	0/34	1/22	6/67	7/123	
6–100	27/34	14/22	42/67	83/123	
>100	0/34	0/22	1/67	1/123	
Not quantified	7/34	7/22	17/67	31/123	
Freckling	23/31	20/20	55/61	98/112	88%
Blue–red macules	1/22	0/12	14/40	15/74	20%
Lisch nodules	3/18	6/15	31/44	40/77	52%
Unilateral	0/18	1/15	2/44	3/77	
Bilateral	2/18	3/15	6/44	11/77	
NS	1/18	2/15	23/44	26/77	
Cutaneous neurofibromas ^b^	2/31	10/19	59/64	71/114	62%
1	0/31	0/19	0/64	0/114	
2–9	2/31	5/19	8/64	15/114	
10–99	0/31	2/19	20/64	22/114	
>100	0/31	0/19	15/64	15/114	
Not quantified	0/31	3/19	16/64	19/114	
Subcutaneous neurofibromas ^b^	3/27	11/14	39/53	53/94	56%
1	2/27	1/14	5/53	8/94	
2–9	1/27	8/14	20/53	29/94	
10–99	0/27	2/14	10/53	12/94	
>100	0/27	0/14	0/53	0/94	
Not quantified	0/27	0/14	4/53	4/94	
Deep neurofibromas	2/13	3/7	15/28	20/48	42%
Plexiform neurofibromas	0/14	3/12	14/49	17/75	23%
Spinal neurofibromas	0/5	3/5	10/25	13/35	37%
Symptomatic	0/5	1/5	5/25	6/35	
Asymptomatic	0/5	1/5	4/25	5/35	
NS	0/5	1/5	1/25	2/35	
OPGs ^c^	3/17	4/16	2/47	9/80	11%
Symptomatic OPGs	0/17	2/16	2/47	4/80	
Asymptomatic OPGs	3/17	2/16	0/47	5/80	
Malignancies ^d^	0/19	1/14	9/51	10/84	12%
MPNSTs	0/19	1/14	3/51	4/84	
Musculoskeletal abnormalities ^e^	13/26	14/22	25/59	52/107	49%
Scoliosis	2/26	6/22	17/59	25/107	
Hyperflexibilty of joints	6/26	3/22	1/34	10/82	
Pectus abnormalities	5/26	2/22	2/34	9/82	
Dysmorphism ^f^	14/24	7/14	8/41	29/79	37%
Short stature (<2 sd)	1/22	2/14	0/41	3/77	4%
Tall stature (>2 sd)	0/22	1/14	0/41	1/77	1%
Macrocephaly (>2 sd)	2/17	0/8	11/33	13/58	22%
Overweight (BMI > 30)	0/20	0/12	7/38	7/70	10%
Neurological abnormalities ^g^	17/23	14/14	29/41	60/78	77%
UBOs	14/23	10/14	11/25	35/62	
Cognitive impairment and/or learning disabilities	22/28	19/21	33/53	74/102	73%
Cardiovascular abnormalities ^h^	6/16	1/11	8/37	15/64	23%
Pulmonic stenosis	1/16	1/11	0/12	2/39	

BMI: body mass index; CALS: café-au-lait spots; MPNSTs: malignant peripheral nerve sheath tumors; NS: not specified; OPGs: optic pathway gliomas; sd: standard deviation; UBOs: unidentified bright objects. * *n* = number of patients fulfilling the criteria; *N* = total number of patients for which data was provided. ^a^ CALS of any size. ^b^ Including non-histologically confirmed cutaneous and subcutaneous neurofibromas. ^c^ OPGs identified with cranial MRI or CT examination. ^d^ Malignancies included MPNSTs (*n* = 4), breast cancer (*n* = 3), non-Hodgkin lymphoma (*n* = 1), intestinal tumor (*n* = 1) and thoracic synovial sarcoma (*n* = 1). ^e^ Musculoskeletal abnormalities included scoliosis (*n* = 25), hyperflexibilty of joints (*n* = 10), pectus abnormalities (*n* = 9), hypotonia/muscle hypotrophy (*n* = 7), feet malformations (*n* = 5), pseudarthrosis (n = 3), osteoporosis (*n* = 3), genu valgum (*n* = 3), large hands/feet (*n* = 2), vertebral dysplasia (*n* = 2), kyphosis (*n* = 2), hyperlordosis (*n* = 1), valgus forearm (*n* = 1). ^f^ When detailed description was available, dysmorphic features included hypertelorism (*n* = 10), low-set ears (*n* = 10), webbed neck (*n* = 6), ptosis (*n* = 5), midface hypoplasia (*n* = 4), downslanting palpebral fissures (*n* = 2), fleshy lips with hypoplastic uvula (*n* = 1), bulbous (*n* = 1) or hooked nose (*n* = 1), strabismus (*n* = 1), coarse (*n* = 1) or triangular face (*n* = 1), low hairline (*n* = 1), Pierre-Robin sequence (*n* = 1). ^g^ Neurological abnormalities included UBOs (*n* = 34), headaches (*n* = 20), epilepsy (*n* = 6), dysplastic cerebellar lesions (*n* = 1), increased visibility of perivascular spaces (*n* = 1), neuro-epithelial cyst in right choroidal fissure (*n* = 1), optic nerve enlargement (*n* = 1), cervical cyst (*n* = 1), vertebral bone matrix abnormalities with dural ectasia and arachnoid cyst (*n* = 1), right pallidum hamartoma (*n* = 1), psychotic episodes (*n* = 1). ^h^ Cardiovascular abnormalities included hypertension (*n* = 7), pulmonic stenosis (*n* = 2), aortic stenosis (*n* = 1), extra-dural hematoma (*n* = 1), patent ductus arteriosus (*n* = 1), septal hypertrophy (*n* = 1), aberrant vessel in ascending aorta (*n* = 1), occlusion of the left internal carotid artery (*n* = 1).

**Table 2:** Comparison of the main clinical findings in the French deleted cohort with “classic” NF1.

Clinical Features	French Deleted Cohort ^a^	“Classic” NF1 Phenotype ^a,b^	*p* Value	Adjusted *p* Value
>5 CALS	84/92 ^c^ (91%)	1537/1728 (89%)	0.61	0.64
Skinfold freckling	98/112 (88%)	1403/1667 (84%)	0.42	0.50
Lisch nodules	40/77 (52%)	729/1237 (59%)	0.24	0.33
Cutaneous neurofibromas ^d^	59/64 (92%)	656/723 (91%)	0.82	0.82
Subcutaneous neurofibromas ^d^	39/53 (74%)	297/515 (58%)	0.028 ↗	0.062
Plexiform neurofibromas ^e,f^	17/61 (28%)	120/648 (18%)	0.089	0.13
Symptomatic spinal neurofibromas	6/35 (17%)	36/2058 (2%)	4.8 × 10^−5^ ↗	1.7 × 10^−4^ ↗
Symptomatic OPGs	4/80 (5%)	64/1650 (4%)	0.55	0.62
Asymptomatic OPGs	5/80 (6%)	70/519 (13%)	0.07	0.13
Malignancies ^g^	10/84 (12%)	18/523 (3%)	0.0024 ↗	0.0072 ↗
Skeletal abnormalities ^h^	42/107 (39%)	144/948 (15%)	2.3 × 10^−8^ ↗	2.1 × 10^−7^ ↗
Scoliosis ^d^	17/59 (29%)	51/236 (22%)	0.30	0.38
Dysmorphism	29/79 (37%)	42/389 (11%)	1.1 × 10^−7^ ↗	6.3 × 10^−7^ ↗
Pulmonic stenosis	2/39 (5%)	25/2322 (1%)	0.072	0.13
Short stature	3/77 (4%)	109/684 (16%)	0.0033 ↘	0.0086 ↘
Macrocephaly	13/58 (22%)	239/704 (34%)	0.082	0.13
Cognitive impairment and/or learning disabilities	74/102 (73%)	190/424 (45%)	5.0 × 10^−7^ ↗	2.2 × 10^−6^ ↗
Cardiovascular abnormalities	15/64 (23%)	54/2322 (2%)	9.2 × 10^−11^ ↗	1.7 × 10^−9^ ↗

Two-tailed exact Fisher test was applied to compare proportions of affected individuals in the two samples. *p* values were adjusted using the Benjamini-Hochberg (B-H) correction for multiple comparisons. Up (↗) and down (↘) arrows respectively indicate a significant increase and decrease in the prevalence of the features in the deleted group versus the “classic” NF1 group at a 5% threshold. CALS: café-au-lait spots; OPGs: optic pathway gliomas. ^a^ Number of patients fulfilling the criteria and total number of patients for which information was provided. ^b^ From Koczkowska et al. 2020 [31] and Pasmant et al. 2010 [34]. ^c^ When excluding the 31 patients for whom CALS were reported but without quantitative data. ^d^ Individuals >18 years old. ^e^ Individuals >8 years old. ^f^ All plexiform neurofibromas in our group, visible plexiform neurofibromas in “classic” NF1 group. ^g^ Including MPNSTs, breast cancer, non-Hodgkin lymphoma, intestinal tumor, and thoracic synovial sarcoma. ^h^ Only including skeletal abnormalities, i.e., scoliosis, pectus abnormalities, feet malformations, pseudarthrosis, osteoporosis, genu valgum, large hands/feet, vertebral dysplasia, kyphosis, hyperlordosis, valgus forearm.

## Data Availability

Data are contained within the article or Supplementary Materials and the crude data are available on request.

## Supplementary Materials

The following are available online at https://www.mdpi.com/article/10.3390/cancers13122963/s1, Figure S1: Violin plot showing the distribution of ages in the deleted cohort, Figure S2: Radar plot for the fulfilling of the 6 clinical NIH criteria in the deleted cohort, Table S1: Main clinical findings in patients with type-1, 2 and 3 *NF1* deletions in the French NF1 cohort and comparison with “classic” NF1, Table S2: Summary of clinical findings and statistical analysis versus “classic NF1” population from previously published and current genotype–phenotype correlations in NF1.
