# Supplementary material for: Severe Phenotype in Patients with Large Deletions of NF1
Source: Cancers (Basel). 2021 Jun 13;13(12):2963. doi: 10.3390/cancers13122963 (PMC8231977; doi:10.3390/cancers13122963)
Supplement: Supplementary file 1 [file cancers-13-02963-s001.zip › cancers-1249603-supplementary.pdf]

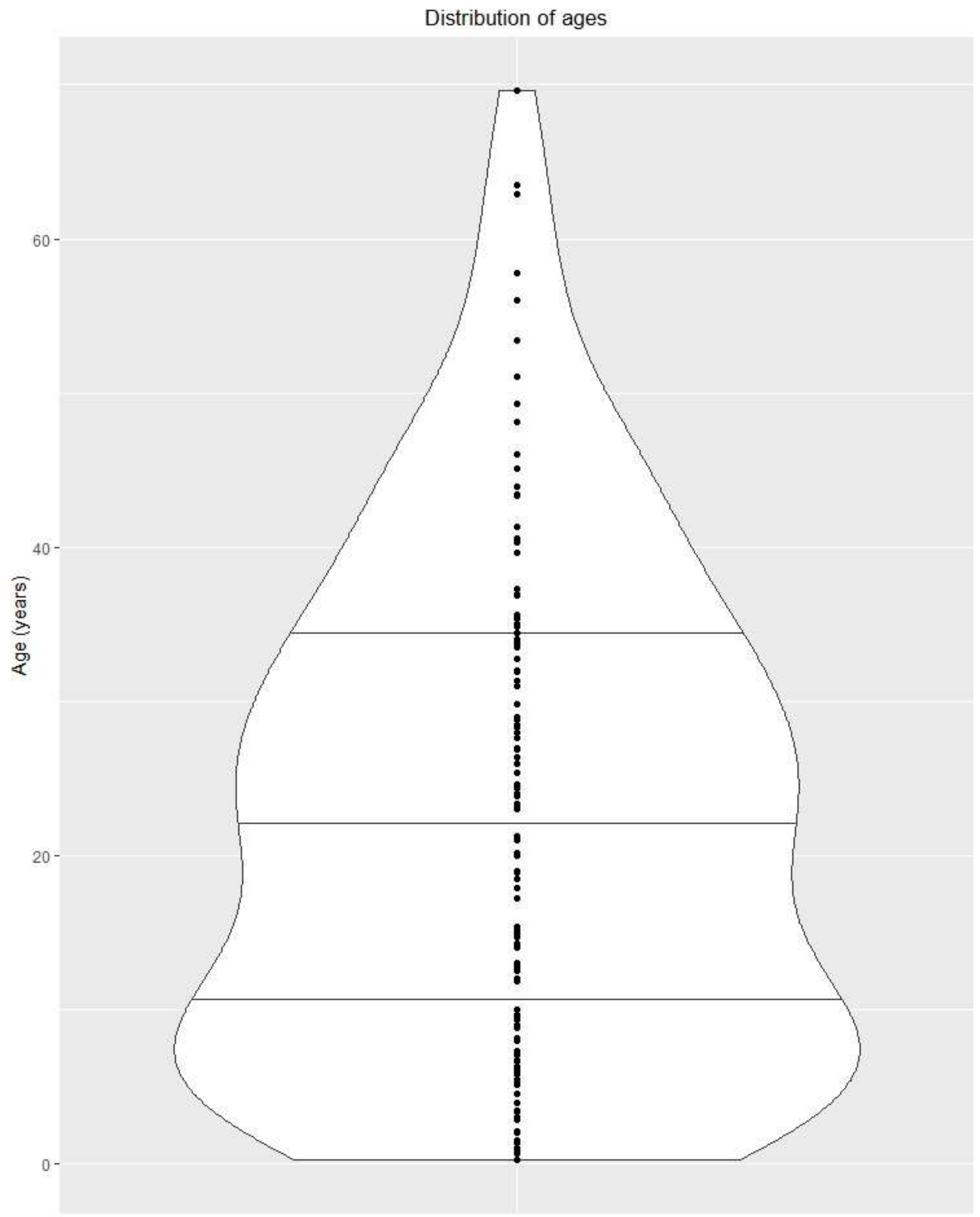

Figure S1. Violin plot showing the distribution of ages in the deleted cohort. Median, first and third quartiles are indicated with full lines. Each dot represents one patient.

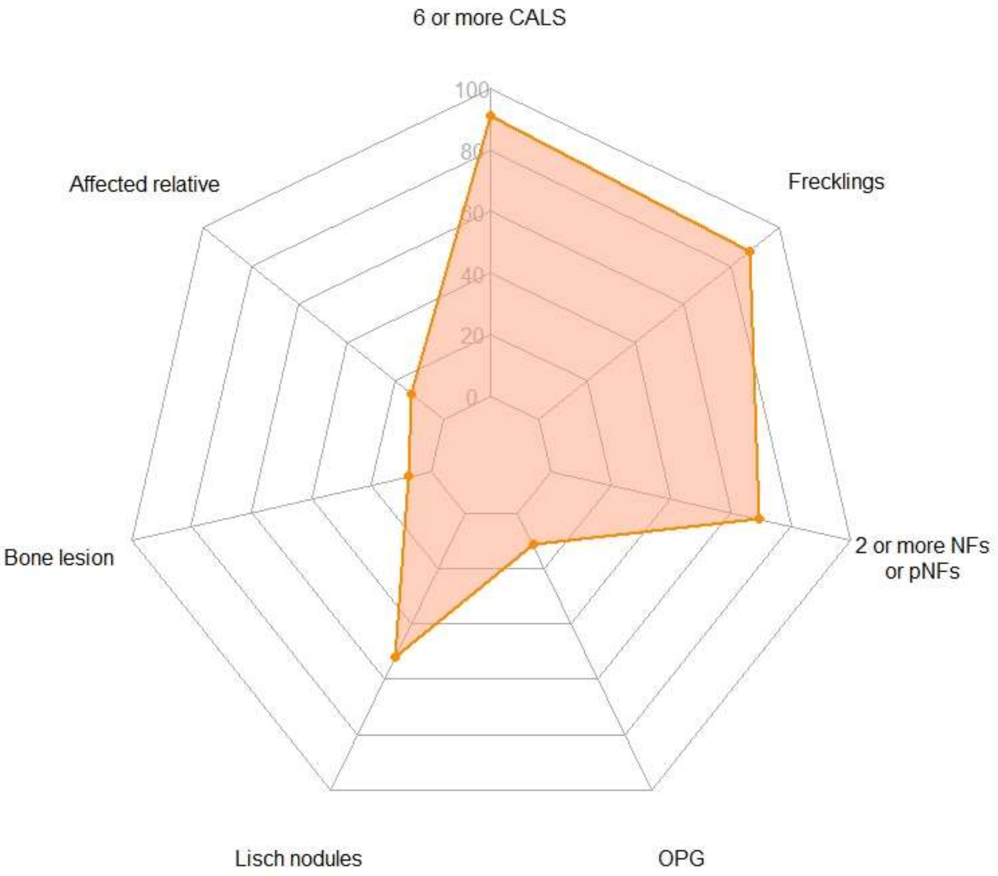

Figure S2. Radar plot for the fulfilling of the 6 clinical NIH criteria in the deleted cohort. CALS: café-au-lait spots; OPG: optic pathway glioma; pNFs: plexiform neurofibromas.

Table S1. Summary of the main clinical findings in patients with type-1, 2 and 3 *NF1* deletions in the French NF1 cohort and comparison with "classic" NF1.

Clinical features	Type-1 deletion	Type-2 deletion	Type-3 deletion	"Classic" NF1 phenotype[a,b]
Age range	8 months-56 yo	1-64 yo	5-41 yo	
Median age (years)	18	24	17,3	
Number of individuals	71	21	5	
Male:Female	30:41	9:12	2:3	
> 5 CALS	50/58 (86%)	8/13 (62%)	3/3 (100%)	1,537/1,728 (89%)
Skinfold freckling	54/62 (87%)	20/20 (100%)	4/4 (100%)	1,403/1,667 (84%)
Lisch nodules	23/44 (52%)	9/15 (60%)	3/4 (75%)	729/1,237 (59%)
Cutaneous neurofibromas[d]	31/31 (100%)	14/18 (78%)	2/2 (100%)	656/723 (91%)
Subcutaneous neurofibromas[d]	24/28 (86%) ↗	9/15 (60%)	1/1 (100%)	297/515 (58%)
Plexiform neurofibromas[e,f]	13/32 (41%) ↗	1/13 (8%)	1/3 (33%)	120/648 (18%)
Symptomatic spinal neurofibromas	2/23 (9%)	1/4 (25%)	1/2 (50%)	36/2,058 (2%)
Symptomatic OPGs	2/43 (5%)	1/16 (6%)	0/5 (0%)	64/1,650 (4%)
Asymptomatic OPGs	2/43 (5%)	0/16 (0%)	1/5 (20%)	70/519 (13%)
Malignancies[g]	4/46 (9%)	1/16 (6%)	0/3 (0%)	18/523 (3%)
Skeletal abnormalities[h]	30/70 (43%) ↗	3/17 (18%)	4/4 (100%)	144/948 (15%)
Scoliosis[d]	10/29 (34%)	3/17 (18%)	2/2 (100%)	51/236 (22%)
Dysmorphism	22/50 (44%) ↗	0/11 (0%)	2/5 (40%)	42/389 (11%)
Pulmonic stenosis	2/30 (7%)	0/3 (0%)	0/1 (0%)	25/2,322 (1%)
Short stature	3/49 (6%)	0/10 (0%)	0/4 (0%)	109/684 (16%)
Macrocephaly	8/37 (22%)	1/8 (13%)	1/3 (33%)	239/704 (34%)
Cognitive impairment and/or learning disabilities	50/59 (85%) ↗	4/16 (25%)	4/4 (100%)	190/424 (45%)
Cardiovascular abnormalities	10/41 (24%) ↗	2/9 (22%)	0/2 (0%)	54/2,322 (2%)

Two-tailed exact Fisher test was applied to compare proportions of affected individuals between each deleted group and the "classic" NF1 cohort. p-values were adjusted using the Benjamini-Hochberg (B-H) correction for multiple comparisons. Up (↗) and down (↘) arrows respectively indicate a significant increase and decrease in the prevalence of the features in the deleted group *versus* the "classic" NF1 group at a 5% threshold after correction. CALS: café-au-lait spots; OPGs: optic pathway gliomas.

[a] Number of patients fulfilling the criteria and total number of patients for which information was provided.

[b] From Koczkowska *et al*. 2020 and Pasmant *et al*. 2010.

[c] When excluding the 21 patients for whom CALS were reported but without quantitative data.

[d] Individuals > 18 years old.

[e] Individuals > 8 years old.

[f] All plexiform neurofibromas in our group, visible plexiform neurofibromas in "classic" NF1 group.

[g] Including MPNST, breast cancer, non-Hodgkin lymphoma and thoracic synovial sarcoma.

[h] Only including skeletal abnormalities, i.e., scoliosis, pectus abnormalities, feet malformations, pseudarthrosis, osteoporosis, genu valgum, large hands/feet, kyphosis, hyperlordosis, valgus forearm.

Table S2. Summary of clinical findings and statistical analysis *versus* "classic NF1" population from previously published and current genotype-phenotype correlations in NF1.

	p.Met992del		p.Arg1809	Codons 844-848	p.Met1149	p.Arg1276	p.Lys1423	Deletions	"Classic" NF1 population
	Upadhyaya *et al*. 2007	Koczkowska *et al*. 2019	Rojnueangnit *et al*. 2015[a]	Koczkowska *et al*. 2018	Koczkowska *et al*. 2020	Koczkowska *et al*. 2020	Koczkowska *et al*. 2020	Present study	From Koczkowska *et al*. 2020.
Number of patients [index cases]	47 [21]	135 [103]	136 [98]	162 [129]	69 [50]	119 [101]	93 [86]	126 [122]	
CALS	47/47 (100)	165/182 (90.7)	124/136 (91.2)	130/157 (82.8) ↘	62/69 (90)	111/119 (93.3)	86/91 (95)	84/92 (91)	1537/1728 (89)
Frecklings	30/47 (64)	105/171 (61.4) ↘*	54/80 (68)	104/144 (72.2) ↘*	40/65 (62) ↘*	74/112 (66.1) ↘*	65/85 (76)	98/112 (88)	1403/1667 (84.2)
Lisch nodules	3/38 (8)	16/139 (11.5) ↘*	10/119 (8.4) ↘	42/98 (43) ↘*	3/44 (7) ↘*	19/70 (24) ↘*	31/59 (53)	40/77 (52)	729/1237 (58.9)
cNFs[b]	0/18 (0) ↘	0–1/57 (0–2) ↘*	0/59 (0) ↘	47/69 (68) ↘*	0–3/24 (0–13) ↘*	14/40 (35) ↘*	23/28 (82)	59/64 (92)	656/723 (90.7)
scNFs[b]		0–3/36 (0–8) ↘*	0-5/59 (0-8) ↘	33/65 (51)	0–3/22 (0–14) ↘*	21/37 (57)	13/23 (57)	39/53 (74) ↗	297/515 (57.7)
Externally visible pNFs[c]	0/41 (0) ↘	0/125 (0) ↘*	0/107 (0) ↘	36/92 (39) ↗*	0/42 (0) ↘*	5/64 (8)	14/48 (29)	17/61 (28)	120/648 (18.5)
Symptomatic spinal NFs	1/47 (2)	1/165 (0.6)		13/127 (10.2) ↗*	0/59 (0)	18/97 (19) ↗*	3/65 (5)	6/35 (17) ↗*	36/2058 (1.8)
Symptomatic OPGs		0/170 (0) ↘*	0/119 (0) ↘	12/136 (8.8) ↗*	0/58 (0)	0/97 (0)	1/74 (1)	4/80 (5)	64/1650 (3.9)
Asymptomatic OPGs		1/41 (2) ↘		18/63 (29) ↗*	0/23 (0)	1/48 (2)	6/40 (15)	5/80 (6)	70/519 (13.5)
Malignancies		6/126 (4.8) (brain tumors)		13/139 (9.4) ↗*	0/57 (0)	4/94 (4)	7/77 (9)	10/84 (12) ↗*	18/523 (3.4)
Skeletal abnormalities[d]		30/172 (17.4)		48/144 (33.3) ↗*	15/61 (25)	32/100 (32) ↗*	34/83 (41) ↗*	35/107 (33) ↗*	144/948 (15.2)
Scoliosis[b]	2/20 (10)	7/57 (12)	6/71 (8) ↘	20/64 (31)	2/20 (10)	8/35 (23)	10/27 (37)	17/59 (29)	51/236 (21.6)
Pectus abnormalities	7/45 (16) ↘	9/125 (7.2)§	9/125 (7.2) ↘		8/61 (13)§			9/82 (11)§	
Noonan-like features		19/166 (11.5) ↗*	32/122 (26.2)	10/134 (7.5) ↗	18/62 (29) ↗*	22/106 (20.8) ↗*	24/83 (29) ↗*		57/1683 (3.4)
Pulmonic stenosis	4/47 (9) ↗	8/160 (5) ↗*	13/105 (12.4) ↗	2/113 (1.8)	2/52 (4)	11/92 (12) ↗*	11/76 (14) ↗*	2/39 (5)	25/2322 (1.1)
Short stature	5/47 (11) ↘	16/118 (13.6)	29/82 (35) ↗	15/91 (16)	5/33 (15)	14/80 (18)	21/51 (41) ↗*	3/77 (4) ↘*	109/684 (15.9)
Macrocephaly	4/45 (9) ↘	30/132 (22.7) ↘	20/80 (25)	36/98 (37)	19/45 (42)	24/76 (32)	15/51 (29)	13/58 (22)	239/704 (33.9)
Cognitive impairment / learning disabilities	8/47 (17) ↘	58/176 (33) ↘*	71/127 (55.9)	56/138 (40.6)	31/66 (47)	46/105 (43.8)	36/87 (41)	74/102 (73) ↗*	190/424 (44.8)
Cardiovascular abnormalities					5/52 (10)	22/92 (24) ↗*	19/76 (25) ↗*	15/64 (23) ↗*	54/2.322 (2.3)

Percentages are indicated into brackets. Up (↗) and down (↘) arrows indicate clinical findings that were described respectively significantly more or less frequently in the studied population *versus* the "classic" NF1 population at a 5% threshold. The asterisk (*) indicates parameters that remained significant after correction for multiple testing with the Benjamini–Hochberg procedure, when applied (Koczkowska *et al*. 2018, Koczkowska *et al*. 2019, Koczkowska *et al*. 2020, present study). Clinical features that were described but not compared to "classic" NF1 population are indicated with an §. Reference cohort for "classic" NF1 group can vary between studies.

[a] Reported data included results from Rojnueangnit *et al*. 2015, Nyström *et al*. 2009, Ekvall *et al*. 2014, Pinna *et al*. 2015 and Santoro *et al*. 2015

[b] Patients aged ≥ 19 years old in Rojnueangnit *et al*. 2015, Koczkowska *et al*. 2018, Koczkowska *et al*. 2019, Koczkowska *et al*. 2020 and in present study; ≥ 20 years old in Upadhyaya *et al*. 2007.

[c] Patients aged ≥ 9 years old in Rojnueangnit *et al*. 2015, Koczkowska *et al*. 2018, Koczkowska *et al*. 2019, Koczkowska *et al*. 2020 and in present study; ≥ 10 years old in Upadhyaya *et al*. 2007

[d] Skeletal abnormalities can include scoliosis, kyphosis, pseudarthrosis, pectus abnormalities, sphenoid wing, long bones, hip or vertebral dysplasia, rib, feet or scapula abnormalities, osteoporosis, genu valgum, large hands/feet, kyphosis, hyperlordosis, valgus forearm, or cysts depending on the studies.
